# Type 2M Von Willebrand Disease: A Case Report

**DOI:** 10.7759/cureus.3112

**Published:** 2018-08-06

**Authors:** Sudeshna Ghosh, Venkata Sunil Bendi, Jairam Krishnamurthy, PavanKumar Tandra, Anuhya Kommalapati

**Affiliations:** 1 Biochemistry, Institute of Post Graduate Medical Education & Research, Kolkata, IND; 2 Neurological Sciences, University of Nebraska Medical Center, Omaha, USA; 3 Hematology/Oncology, University of Nebraska Medical Centre, Omaha, USA; 4 Hematology/Oncology, University of Nebraska Medical Center, Omaha, USA; 5 Internal Medicine, University of South Carolina, Columbia, USA

**Keywords:** von willebrand antigen, von willebrand factor, von willebrand disease

## Abstract

Von Willebrand disease (VWD) is the most common inherited bleeding disorder and is divided into three types, namely type 1, type 2 (2A, 2B, 2M, 2N), and type 3. We report a case of a 24-year-old Caucasian woman with a rare variety of type 2M VWD. Her von Willebrand factor versus antigen ratio was 0.44 (normal ratio is greater than 0.7) . She was asymptomatic and hence not treated but followed up regularly. VWD is not life-threatening when treated timely.

## Introduction

Von Willebrand disease (VWD), first described by Erik von Willebrand, is the most common inherited bleeding disorder [1]. Von Willebrand factor (VWF) carries and protects factor VIII (FVIII) from degradation and helps in hemostasis. When there is an injury to the blood vessel, VWF binds to endothelial cells via Group Ib (GIB) receptors helping the platelets adhesion to the damaged endothelium, thus resulting in the platelet activation and a conformational change. Once activated, the release of adenosine diphosphate from the platelets helps in their aggregation via Group IIb-IIIa receptors. VWD is classified as type 1, type 2, and type 3. Type 1 and type 3 are quantitative defects while type 2 is a qualitative defect [2]. Type 2 is further subdivided into type 2A, 2B, 2M, and 2N. Type 2M is an autosomal dominant variant and is characterized by reduced binding of von Willebrand factor to GP Ib, mild bleeding, and normal von Willebrand factor multimers [3]. We report a rare case of VWD type 2M with its diagnosis and management.

## Case presentation

We report a 24-year-old Caucasian woman who presented to the hematology clinic with a history of easy nontraumatic bruising on both thighs and legs. She had a recent history of fall, with bruises on the left hip, bilateral arms, and the lower left quadrant of the abdomen. Her family history was negative for easy bruisability, bleeding, or clotting disorder. She denied any bleeding gums, heavy menstrual blood flow, nasal bleeds, blood in stool, or blood in urine. She also had an unexplained loss of appetite and loss of weight over the last six months. Her medication history included inhaled albuterol as needed. She has not had any surgeries in the past. She smokes a pack of cigarettes per day and drinks a pint of vodka every night as well as three cans of beer per week. She is unmarried and has no children. She is sexually active with male partners. Her maternal grandmother had lung cancer.

The physical examination was unremarkable except for few bruises on bilateral thighs. The computed tomography (CT) scans of the chest, abdomen, and pelvis were ordered to screen for her unexplained weight loss which was unremarkable. The complete blood count, comprehensive metabolic panel, factor VIII, prothrombin time and partial thromboplastin time, D-Dimer, and antithrombin activity were normal. Her von Willebrand factor activity was much lower compared to the antigen (Table 1). From the reports, it was established that her von Willebrand factor activity was 20% (reference range is 40%-163%) whereas the antigen was low normal at 45% (reference range is 45%-150%) and the ratio is less than 0.5 (normal ratio is more than 0.7). Repeat testing in a week consolidated the previous findings with the factor activity and the antigen as less than 19% and 37%, respectively. The reports of her von Willebrand factor multimer analysis were normal. The platelet count was normal.

**Table 1:** Von Willebrand factor activity and von Willebrand factor antigen values during the observation period.

Date	Von Willebrand factor activity (NL: 40%-163%)	Von Willebrand factor antigen (NL: 45%-150%)	Von Willebrand factor versus antigen ratio (NL: >0.7)
9/12/17	20%	45%	<0.7
9/19/17	<19%	37%	<0.7

## Discussion

Von Willebrand disease affects 1%-2% of the population. Von Willebrand factor is a glycoprotein produced by megakaryocytes and endothelial cells. It is stored in the form of multimers in their secretory granules. The size of the multimers is regulated by a metalloproteinase, ADAMTS13. The primary function of VWF is to serve as a carrier molecule for factor VIII and mediate the attachment of platelets to the damaged endothelium [3].

Von Willebrand disease type 1 is due to the partial deficiency of the factor and responsible for 70% of cases [4]. The clinical manifestation of this type is mild post-operative bleeding after tooth extraction, which is a common finding. This autosomal dominant variant has reduced VWF antigen and cofactor, but the multimers are normal. Factor VIII is proportionately reduced but not as much as VWF [5]. In this type, the ratio of VWF activity to antigen is not reduced and is nearly 1. The type 2A, also an autosomal dominant, is due to the abnormal synthesis, defective packaging before its release or due to the defect in the cleavage site of ADAMTS13 resulting in the loss of multimers. There is a reduced binding of platelets. In type 2B, due to the functional mutations in specific domains of VWF, there is an increased binding of VWF-platelets through GP1B alpha which results in a rapid clearance of these aggregates from the circulation. It results in lowering the levels of VWF along with the multimers and can sometimes cause decreased platelet count too [2]. The type 2N, an autosomal recessive variety, is attributed to a reduced binding affinity of VWF to FVIII. VWD type 3, autosomal recessive, is a rare entity and is characterized by complete absence of VWF [4].

The present case is a type of 2M, an autosomal dominant variety with normal, high molecular weight multimers. It is due to the defect in the A1 domain of VWF gene which encodes for the GIb receptor on the platelets or a missense mutation in the A3 domain. These defects lead to reduced binding between platelets and VWF, resulting in decreased platelet adhesion [6]. The ratio of VWF activity to the antigen is less than 0.7 (normal ratio: >0.7) [2]. 

Laboratory tests include von Willebrand factor antigen, VWF, and factor VIII activity. It is necessary to identify the class of VWD as treatment varies with the subtype. It is advisable to give a try with desmopressin before using the blood products for type 1, 2A, 2M, 2N but not for 2B and type 3 [7]. Desmopressin is known to cause thrombocytopenia in type 2B and in type 3 VWF is completely absent. Hence in these two types VWF concentrate is the treatment of choice. For acute bleeding, desmopressin or tranexamic acid can be used, and if available, VWF concentrate can be tried [8].

## Conclusions

This case represents type 2M VWD, a qualitative defect in von Willebrand factor with the ratio of the factor to the antigen being less than 0.7. In this variety, there is defective adhesion of the factor to platelets although the multimers are normal. It is necessary to screen other family members if VWD is diagnosed in a person. Genetic analysis should be carried out in such cases for all the family members. There are several treatment options available. Therefore, if diagnosed in time, this group of diseases is not life-threatening.
